# Combined Effect of Microstructure, Surface Energy, and Adhesion Force on the Friction of PVA/Ferrite Spinel Nanocomposites

**DOI:** 10.3390/nano12121998

**Published:** 2022-06-10

**Authors:** Moustafa A. Darwish, Tatiana I. Zubar, Oleg D. Kanafyev, Di Zhou, Ekaterina L. Trukhanova, Sergei V. Trukhanov, Alex V. Trukhanov, Ahmed Maher Henaish

**Affiliations:** 1 Physics Department, Faculty of Science, Tanta University, Tanta 31527, Egypt; mostafa_ph@science.tanta.edu.eg (M.A.D.); ahmed.henaish@science.tanta.edu.eg (A.M.H.); 2 Laboratory of Magnetic Films Physics, SSPA “Scientific and Practical Materials Research Centre of NAS of Belarus”, 19, P. Brovki Str., 220072 Minsk, Belarus; olegkan96@mail.ru (O.D.K.); el_trukhanova@mail.ru (E.L.T.); truhanov86@mail.ru (A.V.T.); 3 Laboratory of Single Crystal Growth, South Ural State University, 76, Lenin Av., 454080 Chelyabinsk, Russia; 4 Electronic Materials Research Laboratory, Key Laboratory of the Ministry of Education & International Center for Dielectric Research, School of Electronic Science and Engineering, Xi’an Jiaotong University, Xi’an 710049, China; zhoudi1220@gmail.com; 5 Department of Electronic Materials Technology, National University of Science and Technology MISiS, 119049 Moscow, Russia; 6 NANOTECH Center, Ural Federal University, 620002 Yekaterinburg, Russia

**Keywords:** nanocomposites, ferrites, spinel nanoparticles, surface energy, friction

## Abstract

Nanocomposite films based on spinel ferrite (Mg_0.8_Zn_0.2_Fe_1.5_Al_0.5_O_4_) in a PVA matrix were obtained. An increase in the spinel concentration to 10 wt.% caused an avalanche-like rise in roughness due to the formation of nanoparticle agglomerates. The lateral mode of atomic force microscopy (AFM) allowed us to trace the agglomeration dynamics. An unexpected result was that the composite with 6 wt.% of filler had a low friction coefficient in comparison with similar composites due to the successfully combined effects of low roughness and surface energy. The friction coefficient decreased to 0.07 when the friction coefficient of pure PVA was 0.72. A specially developed method for measuring nano-objects’ surface energy using AFM made it possible to explain the anomalous nature of the change in tribological characteristics.

## 1. Introduction

Nanomaterials attract a lot of attention from researchers and developers due to their anomalous optical, thermal, electrical, and magnetic properties, and significant differences from their bulky counterparts [1]. Spinel ferrites are a hybrid functional material with the general formula MFe_2_O_4_ (M = Mg^2+^, Co^2+^, Mn^2+^, Ni^2+^, Zn^2+^) [2,3,4]. Ferrites are essential for many recent technological designs of electrical, magnetic, and microwave devices [5,6,7]. The properties of ferrites are influenced by the method of synthesis, the composition, the microstructure features, and the distribution of cations in the tetrahedral and octahedral sites in the lattice of the ferrite [8,9,10]. The distribution of equilibrium between the spinel structure cations depends on ionic, electronic, and polarizing effects [11,12,13,14].

Polymers, due to their many properties, have a wide variety of uses, in addition to the invention of new applications [15,16,17,18]. One of these polymers is polyvinyl alcohol (PVA), which can be used as a matrix for many materials due to its good properties, and characteristics such as chemical resistance, mechanical and dielectric strength, solubility in many solvents and water, low cost, environmentally friendly nature, thermal-stability, non-toxicity, and capability for film formation via solution casting [19,20,21,22,23,24,25,26,27,28,29].

One of the primary material science trends is developing composite materials based on a polymer matrix with various fillers [30,31,32,33,34]. Composites often exhibit unexpected behaviors and have properties that differ significantly from those of their components [35,36,37]. The addition of various additives to PVA (from metallic, ceramic, or organic nanoparticles) leads to a significant change in the optical [25,28], electrical [22], sorption [38], and thermal [39,40,41] properties compared to pure PVA. The fillers in PVA can also improve composites’ mechanical properties, hardness, elasticity, tensile strength, and adhesion characteristics [42,43,44,45,46,47,48]. There are several papers on the study of the friction of pure PVA films and composites based on PVA. Pure PVA has the following values of coefficient of friction: 0.65 [49], 0.72 [50], or 0.8 [51], depending on synthesis conditions and other technological parameters. As demonstrated by Chen and colleagues, PVA hydrogel is a promising method for producing ultra-low-friction coatings [49,52,53,54].

The main factor that reduces friction (excluding the effect of roughness) is a decrease in surface energy due to a reduction in the number of unsaturated bond atoms. In addition to changing the polymer’s chemical structure, this can be achieved by introducing nano-sized particles. Due to the high specific surface area, nano-sized particles also have a high concentration of unsaturated bonds. However, a combination with a polymer with good adsorption capacity, such as PVA, is capable of self-removal of unsaturated bonds, and dramatically reduces surface energy; this, in turn, can reduce friction, as well as impart hydrophobicity, as in [44,55].

The problem of obtaining a homogeneous composite based on magnetic nanoparticles, including spinel ferrites, is complex; it usually requires specific synthesis techniques for good homogenization of particles in the matrix and rapid polymerization of the matrix. It is believed that the optimal balance of concentration and surface energy of the nano-sized filler will allow a uniform distribution of nanoparticles to be obtained in the matrix [56,57,58,59,60]. However, this assumption has not previously been tested for magnetic nanoparticles, where there is an additional magnetic contribution to the formation of agglomerates.

This article aims to create a functional magnetic composite material based on ferrite spinel Mg_0.8_Zn_0.2_Fe_1.5_Al_0.5_O_4_ in a PVA matrix with excellent magnetic characteristics and an optimal microstructure, as well as to find the optimal filler concentration for the formation of a homogeneous film. The aim was achieved through comprehensive studies of the structure, adhesion, surface energy, and nano-friction of the composite, depending on the filler concentration. An unexpected result was that the synthesized composite with 6 wt.% Mg_0.8_Zn_0.2_Fe_1.5_Al_0.5_O_4_ had a low coefficient of friction in comparison with similar composites due to the successfully combined effect of microstructure (low roughness) and low surface energy.

## 2. Materials and Methods

### 2.1. Synthesis of Mg_0.8_Zn_0.2_Fe_1.5_Al_0.5_O_4_ Nanoparticles

The flash auto-combustion method [61,62] was used for the spinel ferrite nanoparticle preparation. Spinel ferrite has the formula Mg_0.8_Zn_0.2_Fe_1.5_A_l0.5_O_4_ (MZFA). To achieve the desired composition, stoichiometric amounts of magnesium nitrate (Mg(NO_3_)_2_·6H_2_O), zinc nitrate (Zn(NO_3_)_3_·9H_2_O), aluminum nitrate (Al(NO_3_)_3_·9H_2_O), and iron nitrate (Fe (NO_3_)_3_·9H_2_O) were used. Urea CO(NH_2_)_2_ was used as a fuel. All the metal nitrates were mixed with distilled water using glass rods. The mixture solutions were first prepared, and then stirred at 80 °C for 30 min using a hot-plate magnetic stirrer, followed by adding urea to the mixture while stirring until it becomes viscous and the internal ignition took place, forming brown ferrite nanoparticles. The spinal nanoparticles were annealed at 600 °C for 2 h to avoid any foreign phase.

### 2.2. Synthesis of PVA/MZFA Nanocomposite Films

**Figure 1:**
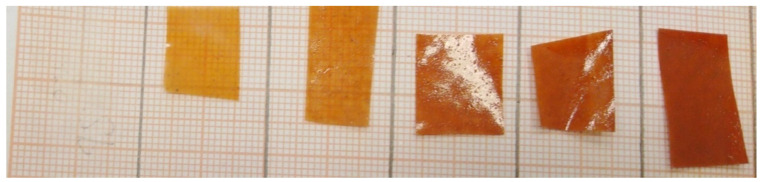
The color gradient of samples with increasing MZFA concentrations. Pure PVA: PVA + 2%MZFA PVA + 4%MZFA PVA + 6%MZFA PVA + 8%MZFA PVA + 10%MZFA.

The PVA/MZFA nanocomposite films were prepared using the solution casting technique. An amount of 10 mg of PVA was dissolved in 200 mL of distilled water with constant stirring with a magnetic stirrer at 70 °C to obtain a clear solution. Various concentrations (2, 4, 6, 8, and 10 wt.%) of MZFA were added to the PVA solution and stirred with a magnetic stirrer at 70 °C for 20 min to obtain a completely homogeneous solution. Then, the mixture of PVA/MZFA was poured into Petri dishes and dried at room temperature. Images of the prepared films are provided in Figure 1.

### 2.3. Characterization

All prepared PVA/MZFA nanocomposite films were characterized using X-ray diffraction (XRD). The patterns were determined using a Shimadzu 600—0XRD X-ray diffractometer using Cu-Kα radiation (λ = 1.54056Å). The pure ferrite sample morphology was investigated using scanning electron microscopy (SEM) (ZEISS Sigma 500 VP FE-SEM), equipped with a secondary electron detector and EDX to quantify the presented elements. Fourier transform infrared (FTIR) spectroscopy was performed using Perkin-Elmer-1430 at room temperature in the range of 200 to 4000 cm^−1^. The microstructure, surface roughness, adhesion force, friction coefficient, and specific surface energy were determined using a multifunctional atomic force microscope (AFM).

### 2.4. AFM Investigations

A multifunctional AFM (NT-2006) was used to investigate sample microstructure, surface roughness, adhesion force, friction coefficient, and specific surface energy. The microscope was designed by ODO “Microtestmachines” (Gomel, Belarus). The scanning speed was 35 µm/s, and the normal load (FN) was about 70 nN. A silicon probe (CSG30 series) with a tip curvature radius of about 10 nm and a force constant of 0.6 N/m was used to obtain the topography. The root-mean-square roughness and average particle size of nanocomposites were calculated using at least five AFM images, as in [63,64]. The following equation estimated the average surface roughness (R_a_):

(1) $$R_{a}=\frac{{ʃ}_{0}^{L}\left|r\left(x\right)\right|\mathrm{dx}}{L},$$

where r(x) is a profile deviation from its mean value and L is a sampling length.

The calculation of particle size was carried out, taking the particle form as an equivalent sphere, as in [65,66]. The adhesion force (F_ad_) was determined using force spectroscopy [67]. The Fad value was equal to the force required to separate the AFM probe from the investigated surface when moving in the normal direction.

The friction at the nano-level was investigated according to the AFM technique. The method of nanofriction investigation with AFM consisted of measuring the torsion angle of the tip under the action of friction forces during contact scanning of the surface in forward and backward directions. It is well described in [68,69,70,71,72]. The value of torsion strongly correlates with the frictional force (F_fr_). Thus, the scan provides information about surface topography (profiles) and profiles of the forward (z_fw_) and backward torsion angle (z_bw_) movements, respectively. Subtracting the backward motion from the forward motion profile gives the resulting graph, which corresponds to the frictional force value. Different ∆Z values characterize the areas with different coefficients of friction and different properties. Roughness zones with different torsion polarities disappear after subtraction. Equation (2) was used for friction force calculation. The friction coefficient (µ) is the ratio of friction force to normal load, as in Equation (3).

(2) $$F_{\mathrm{fr}\;}=\frac{\mathrm{lk}(z_{\mathrm{fw}}-z_{\mathrm{bw}})}{6s\left(1+\vartheta \right)},$$

(3) $$µ=\frac{F_{\mathrm{fr}}}{F_{N}},$$

where l is the cantilever length, k is the stiffness coefficient of the AFM probe, s is the height of the probe tip, ϑ is the Poisson’s ratio of the silicon (material of AFM probe tip), and z_fw_ and z_bw_ are the average cantilever deviations in the forward and reverse motions, respectively.

A unique technique [73] was used to calculate the specific surface energy (SSE) of the nanocomposites. SSE calculation results were obtained from the adapted contact interaction models with the described contact case of the investigated sample using an AFM probe. Equation (4) was used for SSE calculation:

(4) $$\mathrm{SSE}=-\frac{\mathrm{lk}}{6\pi \mathrm{rs}\left(1+\vartheta \right)}\times \left[\int_{0}^{L}\left(f_{\mathrm{fw}}\left(x\right)-F_{\mathrm{fr}}\right)\mathrm{dx}+\int_{L}^{0}\left(f_{\mathrm{bw}}\left(x\right)-F_{\mathrm{fr}}\right)\mathrm{dx}\right]\;,$$

where r is the tip radius, L is the length of the scanning line, and f_fw_ and f_bw_ are functions of the average torsion profiles for the forward and reverse tip movements, respectively.

## 3. Results and Discussion

### 3.1. Structural Analysis

**Figure 2:**
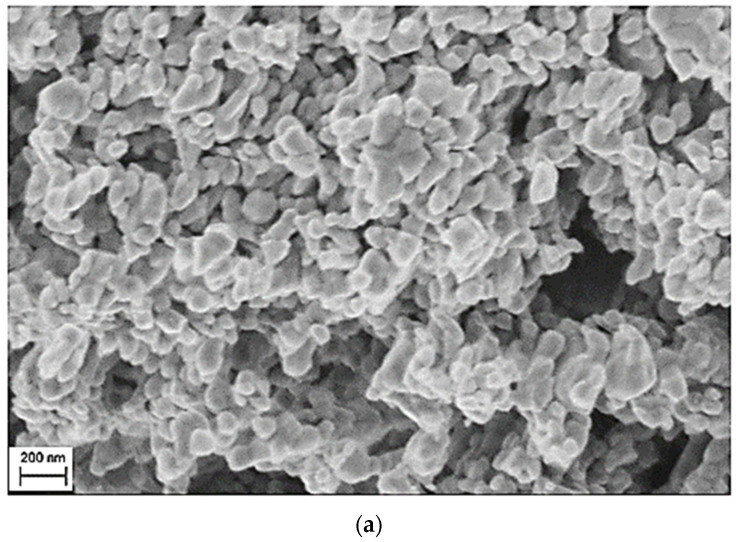

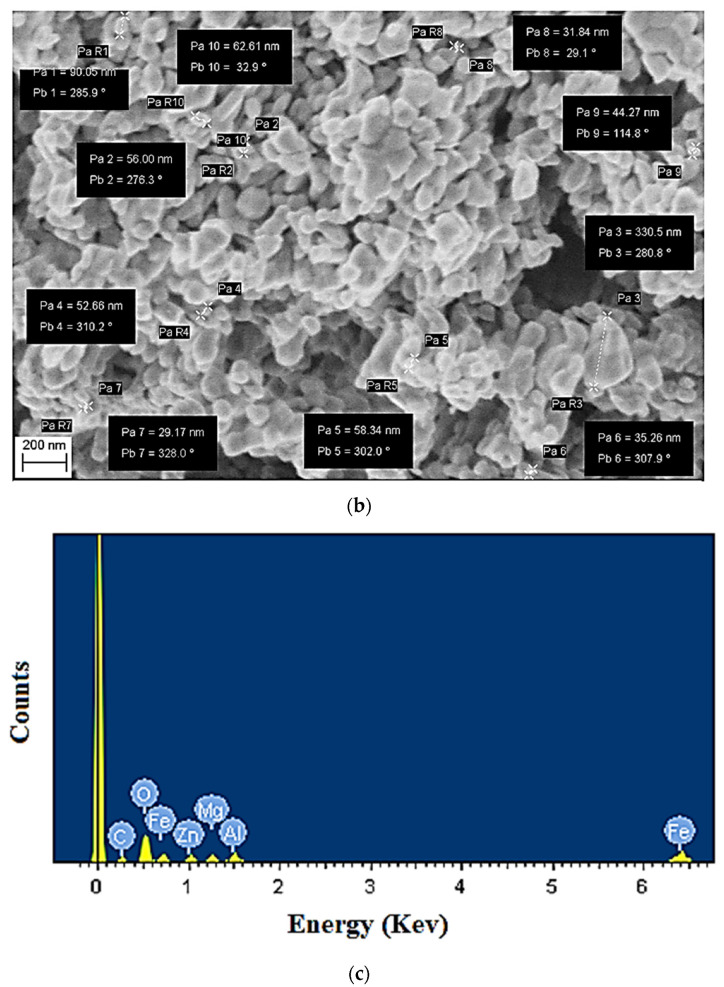
SEM images (**a**,**b**) and EDX analysis (**c**) of the MZFA.

SEM was carried out to illustrate surface morphology, as shown in Figure 2a–c. The average grain size estimated from the micrograph is found to be approximately 51 nm. Elemental analysis employing EDX determines the elemental composition. The estimated stoichiometry is very close to the anticipated values, as given in Table 1.

**Figure 3:**
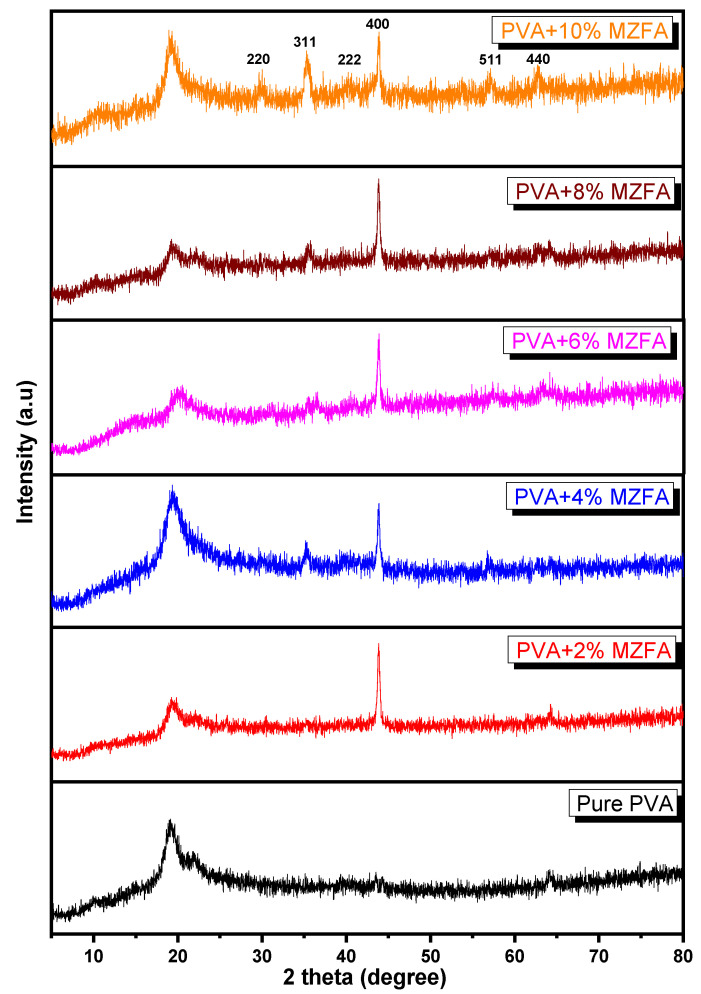
XRD patterns of the pure PVA polymer and synthesized PVA/MZFA composite films.

As shown in Figure 3, the XRD patterns of the pure PVA polymer and the synthesized PVA/MZFA composite films are depicted in the range of 5° ≤ 2θ ≥ 80° at room temperature. It is clearly shown that pure PVA indicates a diffraction band at 2θ = 19.50°, which is attributed to the partially crystalline nature of PVA polymer molecules; this could be as a result of strong intermolecular hydrogen bonding between the PVA chains, although it indicates the presence of a typical semi-crystalline structure [74]. It can be observed that the intensity of the main peak at 2θ = 19.50° is found to decrease with increasing MZFA content in the PVA matrix. This decrease in the intensity may be related to interactions between PVA and MZFA, resulting in an increase in amorphousness due to destroying the PVA chain’s steric regularity [75]. There are additional peaks that appear to confirm that MZFA was present in the PVA matrix, as shown in Figure 3. Furthermore, after doping, the XRD of PVA/MZFA shows new peaks at 29.70°, 35.26°, 43.88°, 57.00°, and 62.72°. The peaks can be indexed to those reflections from the (220), (311), (222), (400), (511), and (440) planes, respectively, and can be attributed to doped ferrite samples.

The interchain separation (R), crystallite size (L), dislocation density (δ), interplanar distance (d), distortion parameters (g), and microstrain (ε) were calculated using the following equations [76,77,78]:

(5) $$R=\frac{5}{8}\frac{\lambda }{\sin\theta },$$

(6) $$L=\frac{k\lambda }{\beta \;\cos\theta },$$

(7) $$\delta =\frac{1}{L^{2}},$$

(8) $$d=\frac{\lambda }{2\;\sin\theta },$$

(9) $$g=\frac{\beta \;}{\tan\theta \;},$$

(10) $$\varepsilon =\frac{\beta \;\cos\theta \;}{4},$$

where θ is the Bragg angle, β is the full width at half maximum (FWHM), k = 0.89, and λ = 1.541178 Å. Table 2 lists the structural parameters (R, L, δ, d, g, and ε) that are calculated relatively to the PVA peak. As shown in Figure 3, the average crystallite size decreases with increasing MZFA content, from 123 to 173 nm, and the dislocation density is inversely proportional to the crystallite size; the value of δ decreases from 6.0 × 10^−2^ to 8.5 × 10^−5^ nm^−2^. A slight change in the value of interplanar and interchain distances is associated with a small change in the angle of the PVA peak. The microstrain and distortion parameters decrease due to the shift in the XRD pattern towards higher 2θ values with increasing MZFA concentration.

According to the analysis in the Williamson–Hall approximation, the contributions to the X-ray peak broadening from the average crystallite size—or in other words, from the average coherent scattering region—and from the microstrain in the crystallite are independent. However, this is true only in the very initial approximation. On closer examination, it becomes clear that the microstrain in a crystallite depends on the average crystallite size. In the literature, one can find results for nanosized particles, for which the interplanar distance decreases with decreasing size [79]. The decrease in the interplanar distance occurs due to compression under the action of the surface tension force. This compressive force is greater the smaller the crystallite size. Such compression leads to an increase in microstrain in the nanocrystallite. Such dependence can be found in [80]. Therefore, a more detailed examination of the relationship between the average crystallite size and microstrain can reveal just the same inverse relationship between these two quantities and, accordingly, contributions to the broadening of the X-ray peak. Each approximation has its drawbacks and, therefore, is not ideal. To prove the applicability of the Williamson–Hall approximation, additional efforts are required. To detail these contributions, it is necessary to use an X-ray diffractometer with an ultrahigh resolution, which is not always available. Nevertheless, in the present work, Formulas (5)–(10) for the relationship between structural parameters that are often used in practice were used. Some examples are the works [76,77,78] and the papers cited herein. The values of the structural parameters will be refined over time.

The asymmetry of X-ray peaks has two explanations. The first explanation is that for the nanosized crystallites, as mentioned above, strong microstrains are observed; these lead to distortion of the unit cell. The symmetry of the obtained nanosized crystallites is no longer strictly cubic as is the case for the massive microsized crystallites of spinel ferrites. Weak distortions, mainly rhombic ones, lead to hardly noticeable splitting of X-ray peaks and their asymmetry for different angular positions. The symmetry of the unit cell in this case can be called pseudocubic rather than ideal cubic. The second explanation for the asymmetry of X-ray peaks is the peculiarities of the size distribution of crystallites. The asymmetry of this dependence also leads to the asymmetry of the X-ray peaks. It should be mentioned that the XRD technique was used to be certain about the phases of the prepared samples and whether there were any foreign phases.

**Figure 4:**
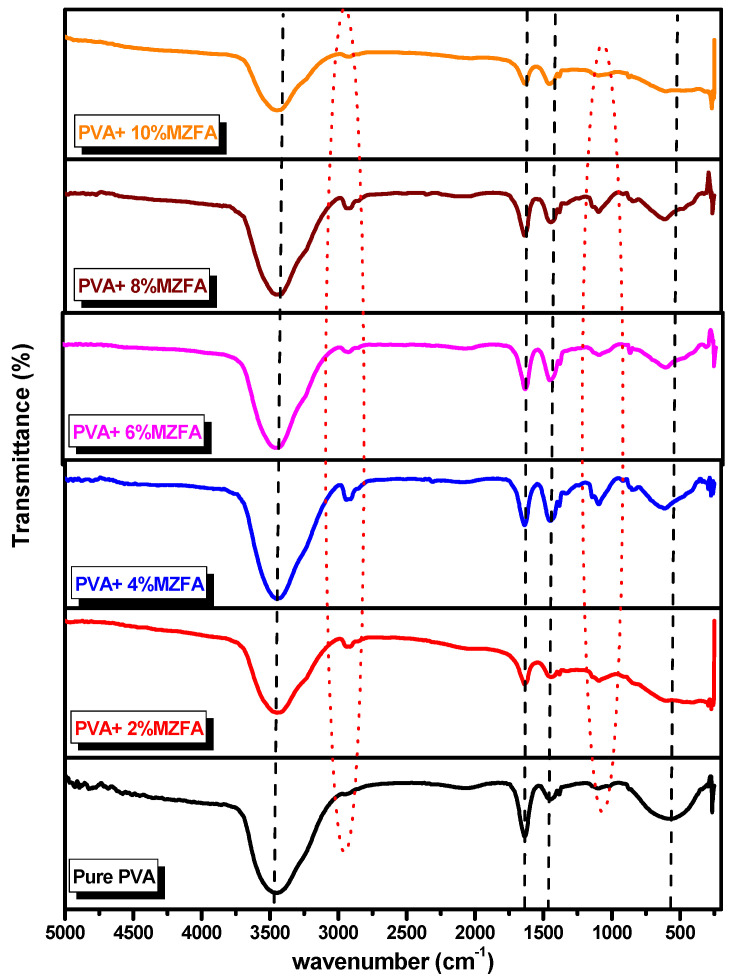
FTIR spectrums of the pure PVA polymer and synthesized PVA/MZFA composite films.

As shown in Figure 4, the FTIR spectra have a range of 200 to 5000 cm^−1^ for all the samples. A broad stretching vibration around 3290 cm^−1^ is observed due to hydroxyl (O-H) groups in the structure of PVA and all the PVA/MZFA composite films. There is a weak band at about 2900 cm^−1^ corresponding to the stretching vibration of CH_2_, implying the presence of PVA. It shows weak bands at about 1630 cm^−1^; additionally, 1460 cm^−1^ corresponds to the H–O–H bending vibration of the residual water, and may be due to stretching vibrations of the anti-symmetric ${\mathrm{NO}}_{3}^{-1}$, respectively. The band around 1090 cm^−1^ is assigned to the vibration of the bond between the oxygen ion and the tetrahedral metal ion O–M_tetra_ [81,82,83]. The dielectric analysis and magnetic properties of the prepared samples will be investigated in detail and provided in the near future.

### 3.2. M. Ultifunctional Atomic Force Microscope (AFM)

**Figure 5:**
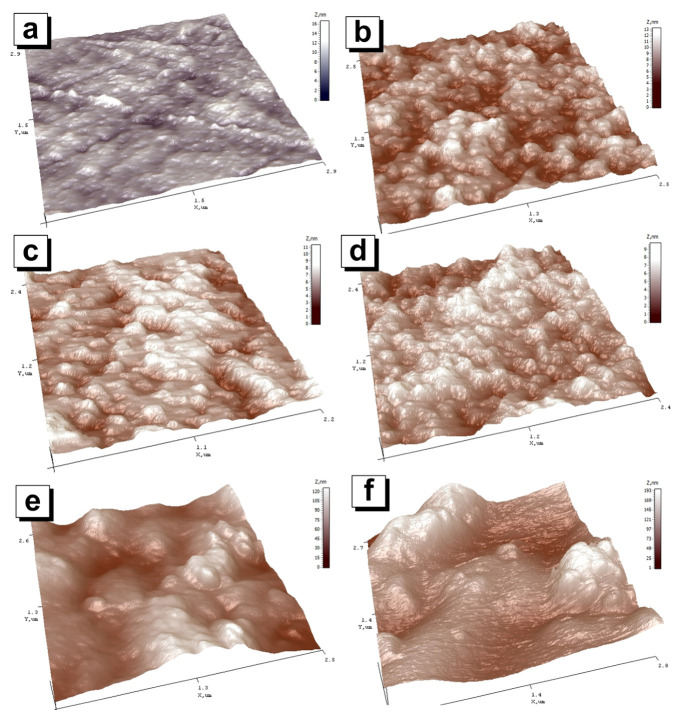
Three-dimensional images of (**a**) PVA matrix and “MZFA/PVA” nanocomposites with nanoparticle concentrations of (**b**) 2 wt.%; (**c**) 4 wt.%; (**d**) 6 wt.%; (**e**) 8 wt.%; and (**f**) 10 wt.%.

**Figure 6:**
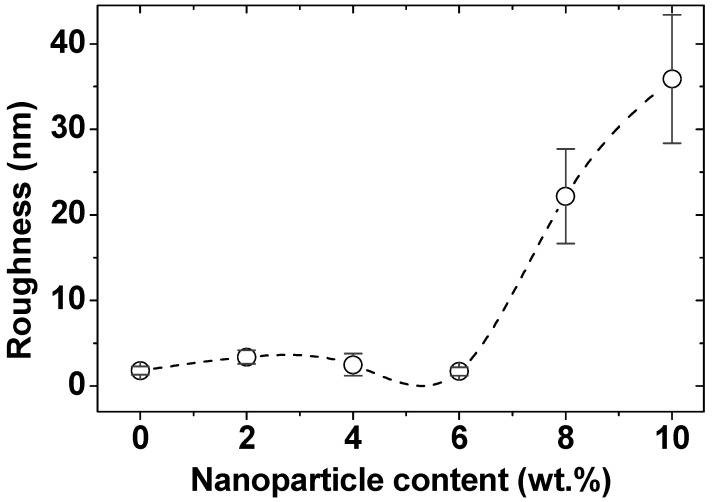
Average surface roughness of nanocomposites.

Figure 5 shows 3D images of composites’ surface topography based on MZFA in a PVA matrix and a matrix without the filler. The matrix surface in Figure 5a is uniform. The height of individual heterogeneity does not exceed a few nanometers. The roughness of the PVA is 1.8 ± 0.5 nm. When 2 wt.% nano-spinel is added to the surface, a distinct grain structure is formed (Figure 5b). The roughness of the composites with a 2–6 wt.% of spinel filler varies in the range from 1.7 ± 0.5 to 3.4 ± 0.8 nm. Figure 6 shows the dependence of the average surface roughness on the amount of filler in the composite. A noticeable increase in roughness begins after the addition of 8 wt.% of filler (until 22.2 ± 5.5 nm). The roughness of the composite with 10 wt.% is 35.9 ± 7.5 nm.

**Figure 7:**
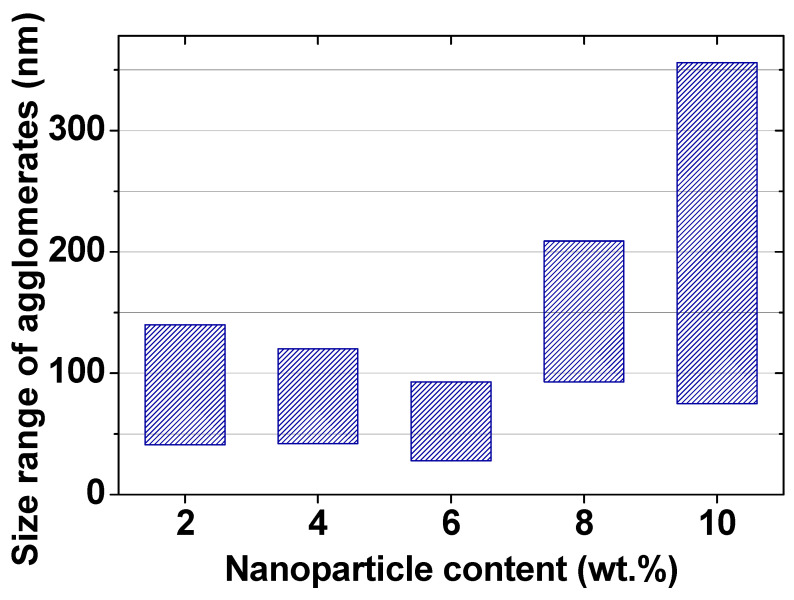
Size range of nanoparticle agglomerates of nanocomposites.

The decrease in the particle size on the surface should also be noted. Figure 7 shows the range of particle sizes for each of the nanocomposites. The visible particles on the surface are probably individual MZFA particles, and their agglomerates are formed during the polymerization of the composite. It can be argued that this is because the same powder with the same particle size was used for all composites, but the visible particle size on the AFM images is different. Estimates of the agglomerate size range from 41 to 140 nm for nanocomposites with 2 wt.% of MZFA, and from 43 to 120 nm and 28 to 93 nm for 4 wt.% and 6 wt.% of nanocomposites, respectively. When the addition of the powder was increased to 8 wt.% and 10 wt.%, the agglomeration process was greatly intensified, as confirmed by the surface topography (Figure 5), the average size of the agglomerates (Figure 7), and the high value of roughness (Figure 6). The roughness graph shows a sharp increase in roughness to 22.2 ± 5.5 nm and 35.9 ± 7.5 nm for samples with 8 wt.% and 10 wt.% filler, respectively. In these nanocomposites, nanoparticle agglomerate size is in a wide range, from 92 to 209 nm (for 8 wt.%) and 75–356 nm (for 10 wt.% of nanocomposite).

**Figure 8:**
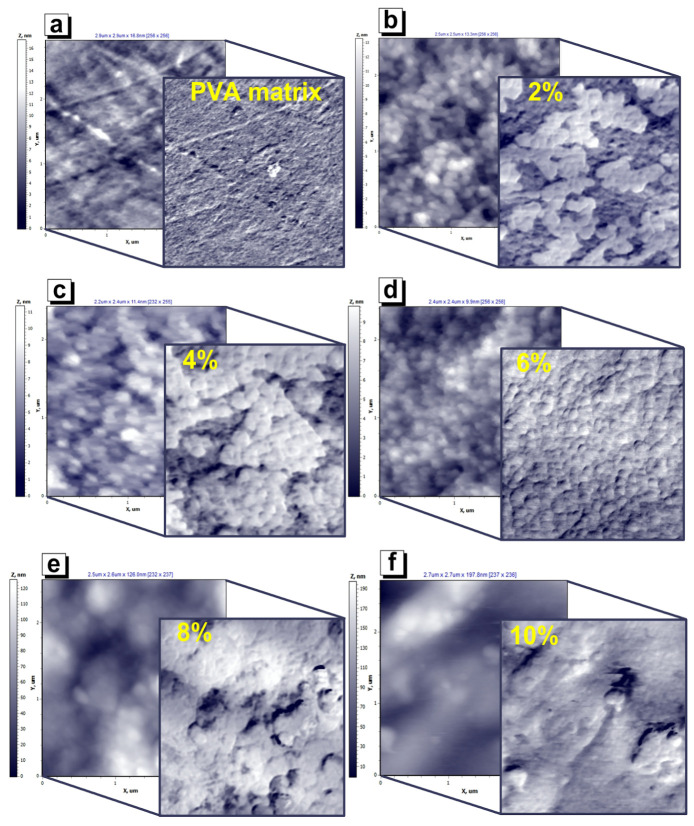
Surface microstructure including topography (bottom images) and lateral forces maps (overhead images) of (**a**) PVA matrix and “MZFA/PVA” nanocomposites with filler concentrations of (**b**) 2 wt.%; (**c**) 4 wt.%; (**d**) 6 wt.%; (**e**) 8 wt.%; (**f**) 10 wt. %.

Figure 8a–f show the 2D image of the microstructure of the pure matrix and nanocomposite surfaces, respectively. There are two images for each sample. The first image (bottom) is the topography, which describes the sample roughness and microstructure. The second image was obtained in the lateral force mode or in the contact mode of the atomic force microscopy peaks. The second image (overhead) demonstrates the magnitude of the forces that twist the AFM probe during scanning. These forces are most-often associated with frictional forces, as they impede the movement of the tip of the probe over the surface under study [84,85,86]. However, friction at the nanoscale has a more complex nature than at the macroscale. At the macroscale, the main contributor to friction is surface roughness. At the nanoscale, the force of adhesion; interatomic and intermolecular interactions (van der Waals force); and electrical, magnetic, and chemical interactions also have a strong effect. The contribution of each of these components to nano-friction processes is poorly understood and little-studied in the literature due to its complex influence. However, it can be asserted, with complete confidence, that individual sections of composite images with a 2, 4, and 8 wt.% of MZFA filler interact with an AFM probe at different strengths. This is confirmed by the contrasting areas in the lateral forces maps’ overhead images for nanocomposite with a 2, 4, and 8 wt.% of filler in Figure 8b,c,e).

The uniformity of the pure matrix surface indicates a high degree of polymerization, as can be seen from Figure 8a. Figure 8b,c are similar, but differ in the ratio of dark-to-light areas. An increase in the powder concentration from 2 wt.% to 4 wt.% causes a rise in the number of light regions compared to dark ones. Light areas are characterized by a greater force of twisting of the probe and, accordingly, by a greater force of interaction than dark areas, by less. The map of lateral forces of the sample with 6 wt.% of filler is uniform. Based on this fact, it can be concluded that a concentration of 6% for the composites is optimal for the formation of a homogeneous nanocomposite. The composite filling degree (particle area percentage) is approximately 45% of the surface for a 2 wt.% concentration, and approximately 70% for a 4 wt.% concentration (Figure 8b,c). The surface looks uniformly filled with MZFA at a concentration of 6 wt.%. However, an unexpected decrease in the filling degree of 90% is observed for the concentration of 8 wt.% of the filler in the PVA matrix, as judged by the lateral force maps in Figure 8e. It is probable that the concentration of 8 wt.% is excessive and causes a “new wave” of agglomeration.

Moreover, bulk agglomerates are formed, which leads to a significant increase in roughness, as noted earlier in Figure 6. The lateral force image for a composite with 10 wt.% of the filler (Figure 8f) does not provide obvious information about the agglomerates’ structure, since there is no visible contrast on the lateral force map. There are two options for this: (1) nano-sized MZFA uniformly filled the empty matrix between the agglomerates; or (2) the formation of sufficiently large (75–356 nm) agglomerates caused a decrease in their surface energy (or energy of the surface and AFM probe interaction) to such an extent that it became equal to the surface energy of the PVA matrix. As a result, the differences in the forces of interaction between the matrix and the filler with the probe disappear.

**Figure 9:**
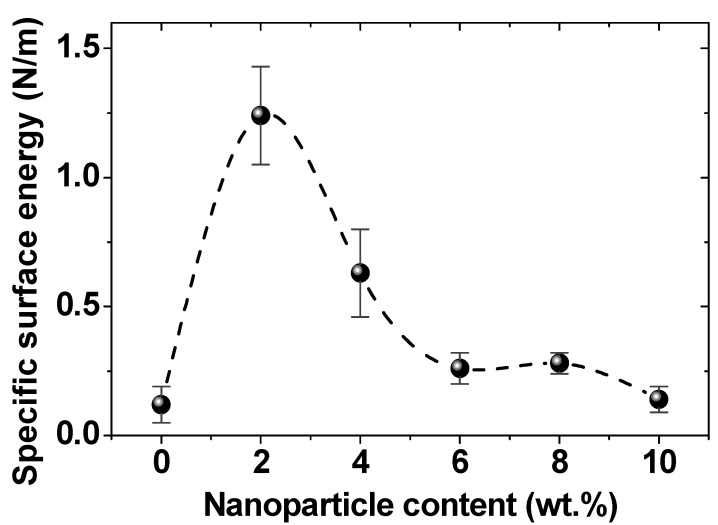
The specific surface energy of “MZFA/PVA” nanocomposites.

Using the previously developed unique technique for determining the specific surface energy at a single point using AFM [73], SSE studies were carried out for “MZFA/PVA” nanocomposites. The measurements were carried out at points coinciding with the center of the individual particles, which are clearly visible in the AFM images. The spinel particles correspond to the light areas in lateral force map images (Figure 8). Studies were conducted on at least 20 points for each sample to reduce the measurement error. A set of at least 20 measurements at random locations was made for pure PVA matrices. The results of the SSE investigations are shown in Figure 9. The SSE of the pure PVA matrix is 0.12 ± 0.07 N/m. The composite with 2 wt.% filler has a surface energy more than ten times higher than the SSE of the pure matrix (1.24 ± 0.19 N/m). SSE is reduced two times each time, with an increase in the filler’s concentration to 4 wt.% and 6 wt.% (to 0.63 ± 0.17 and 0.26 ± 0.06 N/m, respectively). The change in the specific surface energy correlates well with the uniformity of filling the composite with MZFA particles. Thus, the maximum surface energy is observed at the minimum filler concentration, and then SSE decreases and reaches a minimum when the particle distribution in the matrix becomes uniform (6 wt.%). A further increase in filler concentration to 8 wt.% has no significant effect on SSE. The SSE of a composite with 8 wt.% is 0.28 ± 0.04 N/m. However, with an increase in the filler concentration to 10 wt.%, the surface energy is reduced two times compared to the sample with 8 wt.%: SSE = 0.14 ± 0.05 N/m. This confirms the fact that there is a “second wave” of agglomeration, as described above.

**Figure 10:**
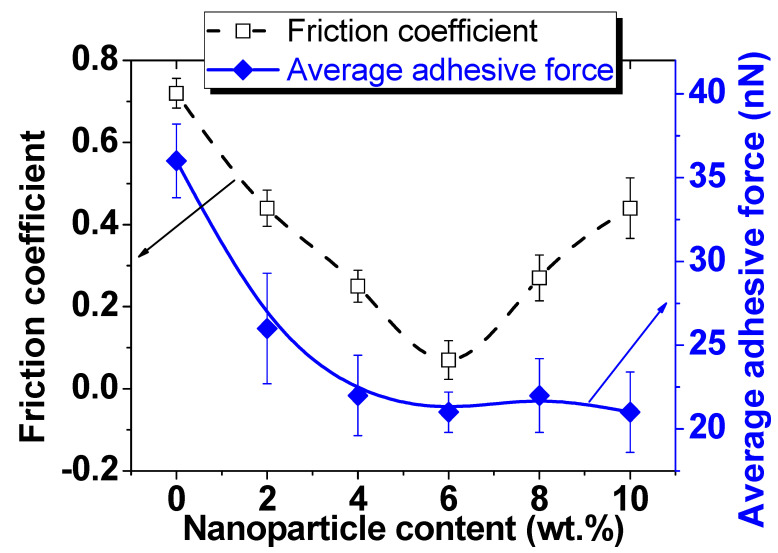
The friction coefficient and average adhesive force of “MZFA/PVA” nanocomposites.

Figure 10 shows the study results of the friction coefficient (black line), which was measured in the contact AFM mode. Usually, the friction force (as well as the friction coefficient) correlates well with the roughness [68,87,88]. In this study, the maximum friction coefficient value (0.72) corresponded to the pure PVA matrix, characterized by the minimum surface roughness (Ra = 1.8 nm). It should be recalled that the pure matrix also has a minimum surface energy (SSE = 0.12 N/m). To explain the anomalously high value of friction, adhesion force studies were carried out using the force spectroscopy method. The results are shown in Figure 9 with a blue line.

It was found that despite the low surface energy, the pure matrix has the highest adhesion force to the probe (36 nN). It should be noted that this refers to the adhesion force of the probe and the sample surface during separation in the normal direction. The addition of 2 wt.%, 4 wt.%, and 6 wt.% of MZFA to the PVA matrix leads to a linear decrease in both the friction coefficient to µ = 0.07 and the adhesion force to F_ad_ = 21 nN for the sample with 6 wt.% filler. As was noted, the value of the friction coefficient depends on the friction force and the normal load (Equation (3)). Since the normal load was kept constant during the measurement process (about 70 nN), only the friction force, which depends on the roughness, adhesion force, and surface energy, contributes. The surface of the PVA/MZFA composite with 6 wt.% of filler has a low roughness (Ra = 1.7 nm, Figure 6), as well as a small distribution of particle sizes (Figure 7), which reduces the number of unsaturated bonds. This results in a low surface energy value (SSE = 0.26 N/m, Figure 9). As a result of the combined influence of these factors, the PVA/MZFA composite with 6 wt.% of filler has a low value of friction force (Ffr = 4.9 nN) and friction coefficient (μ = 0.07). The coexistence of these characteristics makes this material promising as a tribological coating. A further increase in filler concentration does not lead to changes in the adhesion forces, which are 22 ± 2.2 nN (for 8 wt.%) and 21 ± 2.4 nN (for 10 wt.%). However, a sharp increase in the coefficient of friction to 0.27 and 0.44 is observed with an increase in concentration to 8 wt.% and 10 wt.%, respectively. This increase was caused by the increase in surface roughness that is observed for these samples.

## 4. Conclusions

The studies of the surface morphology of nanocomposites and lateral forces maps using AFM methods show that the addition of 6 wt.% of MZFA nano-spinel leads to the formation of a composite with low roughness (Ra = 1.7 nm) and a good uniformity of distribution of nano-spinel particles. Concentrations lower than 6 wt.% are also characterized by low roughness, but the particles are not uniformly distributed. An increase in filler concentration to 8 and 10 wt.% caused intense agglomeration, which increased the roughness to 22.2 and 35.9 nm, respectively. The formation of a homogeneous composite and agglomeration were accompanied by corresponding changes in the specific surface energy, which was measured using a unique, previously developed AFM technique. In addition, it is shown that the friction coefficient of “MZFA + PVA” nanocomposites strongly depends on the adhesion force at low roughness. When the Rq value increases due to agglomeration, roughness determines the tribological characteristics. Thus, it was found that the uniformity of nanoparticle distribution, low roughness, surface energy, and adhesion force characterized the composite with 6 wt.% of MZFA. As a result of the above, this film has an abnormally low value of the friction coefficient (µ = 0.07) for composites including PVA and nanoparticles.

## Figures and Tables

**Table 1:** The results of EDX analysis.

Elements	Mg	Zn	Al	Fe	O	C
Weight %	6.57	5.56	4.82	20.75	43.47	18.83
Atomic %	5.21	1.64	3.44	7.16	52.35	30.21

**Table 2:** The structural parameters of the polymer blend PVA/MZFA composite.

Sample	Crystallite Size (L), (nm)	Interchain Separation (R), (nm)	Interplanar Distance (d), (nm)	Microstrain (ɛ), (arb. un.)	Dislocation Density (δ), (nm^−2^)	Distortion Parameters (g), (arb. un.)
Pure PVA	123	6.2	5.0	8.0 × 10^−2^	6.0 × 10^−2^	2.30
PVA + 2% MZFA	127	3.7	3.0	3.2 × 10^−2^	1.2 × 10^−4^	0.50
PVA + 4% MZFA	165	3.9	3.1	2.7 × 10^−2^	8.7 × 10^−5^	0.43
PVA + 6% MZFA	146	3.8	3.1	3.0 × 10^−2^	1.0 × 10^−4^	0.49
PVA + 8% MZFA	168	4.0	3.2	2.8 × 10^−2^	9.0 × 10^−5^	0.45
PVA + 10% MZFA	173	4.0	3.2	2.7 × 10^−2^	8.5 × 10^−5^	0.47

## Data Availability

Not applicable.
